# Central Nervous System Involvement in Primary Adrenal Non-Hodgkin Lymphoma

**DOI:** 10.4274/tjh.2013.0432

**Published:** 2014-09-05

**Authors:** Padhi Somanath, Sahoo Jayaprakash

**Affiliations:** 1 Pondicherry Institute of Medical Sciences (P.I.M.S), Department of Pathology, Pondicherry, India; 2 Jawaharlal Institute of Postgraduate Medical Education and Research, Department of Endocrinology and Metabolism, Pondicherry, India

**Keywords:** Primary adrenal non-Hodgkin lymphoma, Central nervous system, prognosis, therapy

## TO THE EDITOR

We read with great interest the case report of central nervous system (CNS) involvement in primary adrenal lymphoma (PAL) in an elderly, HIV-seronegative male patient by Aydın et al. in the December issue of your journal [1]. In spite of initial partial regression of the CNS lesion, the patient succumbed to progressive CNS disease after rituximab-based chemotherapy and whole-brain radiotherapy. Though the pathogenesis and therapeutic aspects of this lymphoma at both anatomic sites were highlighted, there was a lack of precise information regarding adrenal function (prior autoimmune adrenalitis) and detailed immunophenotype of PAL (germinal center or non-germinal center), which could have also influenced the clinical outcome in this patient.

PAL is an enigmatic entity with nearly 200 cases reported in the world literature up to 2013 [2,3]. Out of all parameters studied, adrenal insufficiency, high lactate dehydrogenase (LDH), B symptoms, and initiation of chemotherapy have been reported to be the significant independent predictors of poor prognosis in PAL. Secondary CNS involvement is known to occur in 2%-10% cases of diffuse large B-cell lymphoma (DLBCL) and confers a poor prognosis [3]. Of all reported cases of PAL (1980-2013, including the case by Aydın et al.), 18 patients had CNS involvement [7 (39%) at presentation, 11 (61%) at relapse (within 6 months of diagnosis)]. Their mean age was 63.8 years (range: 42 to 82 years), 17/18 (94.5%) were male, 16/16 (100%) had bilateral PAL, 10/13 (77%) had a mean lesion size of 5 cm or more, 3/18 (16.6%) had disseminated disease at presentation, 1/18 (5.5%) had coexistent secondary involvement of thyroid, 9/11 (82%) had adrenal insufficiency, 11/13 (84.6%) had elevated LDH, and 11/14 (78.5%) had B symptoms. Thirteen of 18 (72%) had DLBCL, 2 had peripheral T-cell lymphoma, 1 had Burkitt-like lymphoma, and the remaining 2 (11%) had non-Hodgkin lymphoma unclassified [2,3] (Table 1). Though patients with PAL are at risk of CNS involvement, there has been no consensus, at present, regarding CNS-directed prophylaxis in these patients. As most of the reported CNS events in PAL cases occurred prior to the rituximab era, larger in-depth prospective studies in the post-rituximab era will, hopefully, throw more light on this topic in future.

## CONFLICT OF INTEREST STATEMENT

The authors of this paper have no conflicts of interest, including specific financial interests, relationships, and/ or affiliations relevant to the subject matter or materials included.

## Figures and Tables

**Table 1:**
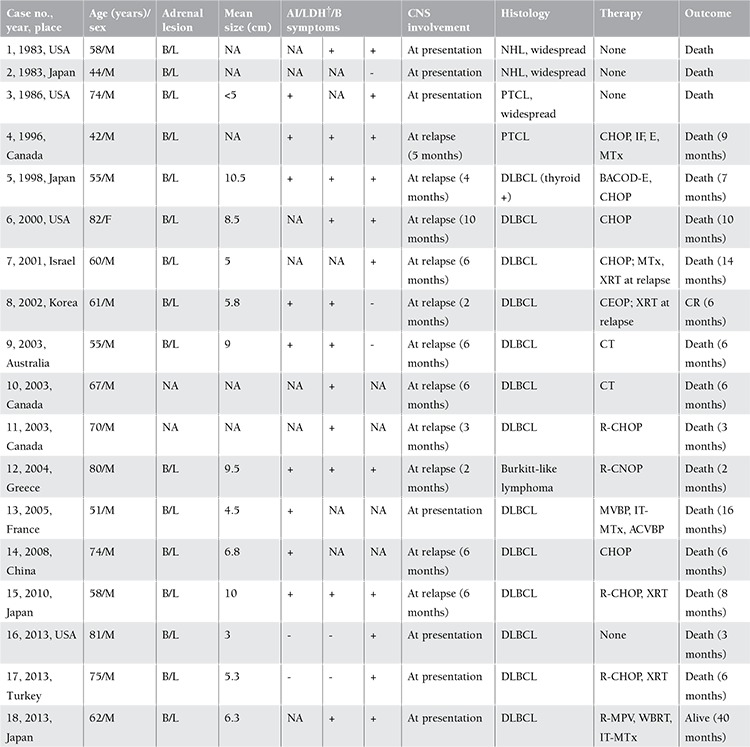
Central nervous system involvement in primary adrenal non-Hodgkin lymphoma (PAL): review of literature (1980-2013, n=18).
